# Numerical simulation and analysis of effects of individual differences on the field distribution in the human brain with electromagnetic pulses

**DOI:** 10.1038/s41598-021-96059-3

**Published:** 2021-08-13

**Authors:** Shan Wang, Zhongguo Song, Huiping Li, Guozhen Guo, Xiaoli Xi

**Affiliations:** 1 Faculty of Automation and Information Engineering, Xi’an University of Technology, Xi’an, 710048 China; 2 Department of Radiation Biology, Air Force Medical University, Xi’an, 710032 China

**Keywords:** Computational biophysics, Biological physics, Computational models, Biophysics

## Abstract

The blood–brain barrier (BBB) opening induced by electromagnetic pulses (EMPs) may be a drug delivery strategy of central nervous system (CNS) diseases. However, the mechanism of EMP-induced BBB opening is still ambiguous. Previous studies have shown the relation between the external field and the extent of BBB permeation (referred to as the effect), while the connection between the internal field and the effect remains unknown. Here, the influence of individual differences on the field distribution in the human brain with EMPs is investigated, the dielectric parameters of the specific anthropomorphic mannequin (SAM) and structural parameters of the spherical brain are adjusted, and the field distribution in the brain illuminated by EMPs at the frequency range of 0–0.5 GHz is simulated based on the Computer Simulation Technology (CST) Studio Suite. The results show that the average electric field in the brain is about 1/100–1/5 of the incident field within the studied frequency range, individual differences have little effect on the field distribution in the human brain; and thus, it is reliable to establish the connection between the internal field and the effect, which is of great theoretical significance for further study of the mechanism of an EMP on the brain.

## Introduction

With the aggravation of environmental pollution and the ageing of the population, central nervous system (CNS) diseases have become a major public health issue^1,2^. Meanwhile, an increasing incidence of CNS diseases has been reported from multiple studies^3–8^. Much effort has been made to develop therapeutics for these diseases. However, a big clinical challenge is that drugs cannot be effectively delivered to the CNS due to the existence of the blood–brain barrier (BBB), the main obstacle^9,10^. Previous studies have shown that an electromagnetic pulse (EMP) could open BBB locally, transiently and reversibly^11–13^, which is expected to provide a new avenue for the electromagnetic diagnosis and treatment of these CNS diseases. However, the mechanism of EMP-induced BBB opening is still ambiguous till now.

In the last decades, many studies only showed an apparent relation between the external field and the extent of BBB permeation (referred to as the effect) based on animal experiments^12,13^. The research on the physical connection between the internal field and the effect has not been developed, and which is very important to study the mechanism of EMP-induced BBB opening. However, studies on the influence of individual differences (individual dielectric parameters differences and structure differences) on the field distribution in the brain is essential to establish the connection between the internal field and the effect. Lin^14,15^ considered pulsed-wave propagation in spherical models and determined the transmitted field strengths in homogeneous spherical models of human and animal heads, but the influence of certain input parameters on the modeled outputs remains ambiguous. Moreover, in terms of individual dielectric parameters differences, Gabriel^16–18^ investigated 20 kinds of human and animals’ tissues, and found that the variation range of dielectric parameters was about ± 5–10%. Gabriel^19,20^ also attached great importance to the change of dielectric parameters of biological tissues with age and found that the dielectric parameters variation range in tissues was ± 1% to ± 10% and ± 15% to ± 25%. In terms of individual structure differences, the specific anthropomorphic mannequin (SAM) model was scaled to 80 to 100% sized models at intervals of 5% to investigate the relation between the local specific absorption rate (SAR) and head size in a previous study^21^. Wang^22^ studied preliminarily the influence of individual differences on the internal field distribution of the brain exposed to EMPs, but the effect of individual differences on the field distribution at various points in the brain has not been considered.

In order to study the influence of individual differences on the field distribution under the radiation of an EMP, the electromagnetic distribution in the brain is the main concern of this paper. Therefore, it is necessary to construct a suitable physical model for each part of the human brain. Although, many high-resolution anatomical body models exist that include the dielectric properties of individual organs and tissues, it is difficult to rapidly estimate the uncertainties caused by varying certain parameters within the confounding effects of comparing results against the anatomically accurate phantoms.

In this study, simplified cases including the SAM and a spherical brain phantom were adopted to simulate an EMP incident on a human brain with the Computer Simulation Technology (CST) Studio Suite 2016^23^. On the one hand, the typical SAM model is used and its dielectric parameters are varied. Considering the fact that dielectric properties fluctuate in a certain range due to individual health and age status, in this work, according to Gabriel^16–20^, each dielectric parameter will be increased or decreased by 10% or 20%, respectively. On the other hand, the spherical brain model is constructed, and different structural parameters are given. The structural parameters of human brain are related to the race, sex, age, and so on, taking that into account, in this paper, the head size and skull thickness will be increased or decreased by 10% or 20%, respectively (Reference^21^). The field distribution in the phantoms was calculated and analyzed to assess the influence of individual differences on the electromagnetic distribution, which would build some modeling datasets with respect to model outcomes while varying certain parameters like the dielectric properties. The datasets are useful for characterizing the uncertainty of the fields following EMP pulses (referred to as the uncertainty quantification) and the uncertainty quantification (UQ) data from these simulations can inform further studies that utilize high-fidelity models, where the high-fidelity results can be used to corroborate the findings here, and add additional data to the overall UQ. Also, it is of great theoretical significance for further research of the action mechanism of an EMP on the brain.

## Models and methods

### SAM and spherical brain models

**Figure 1:**
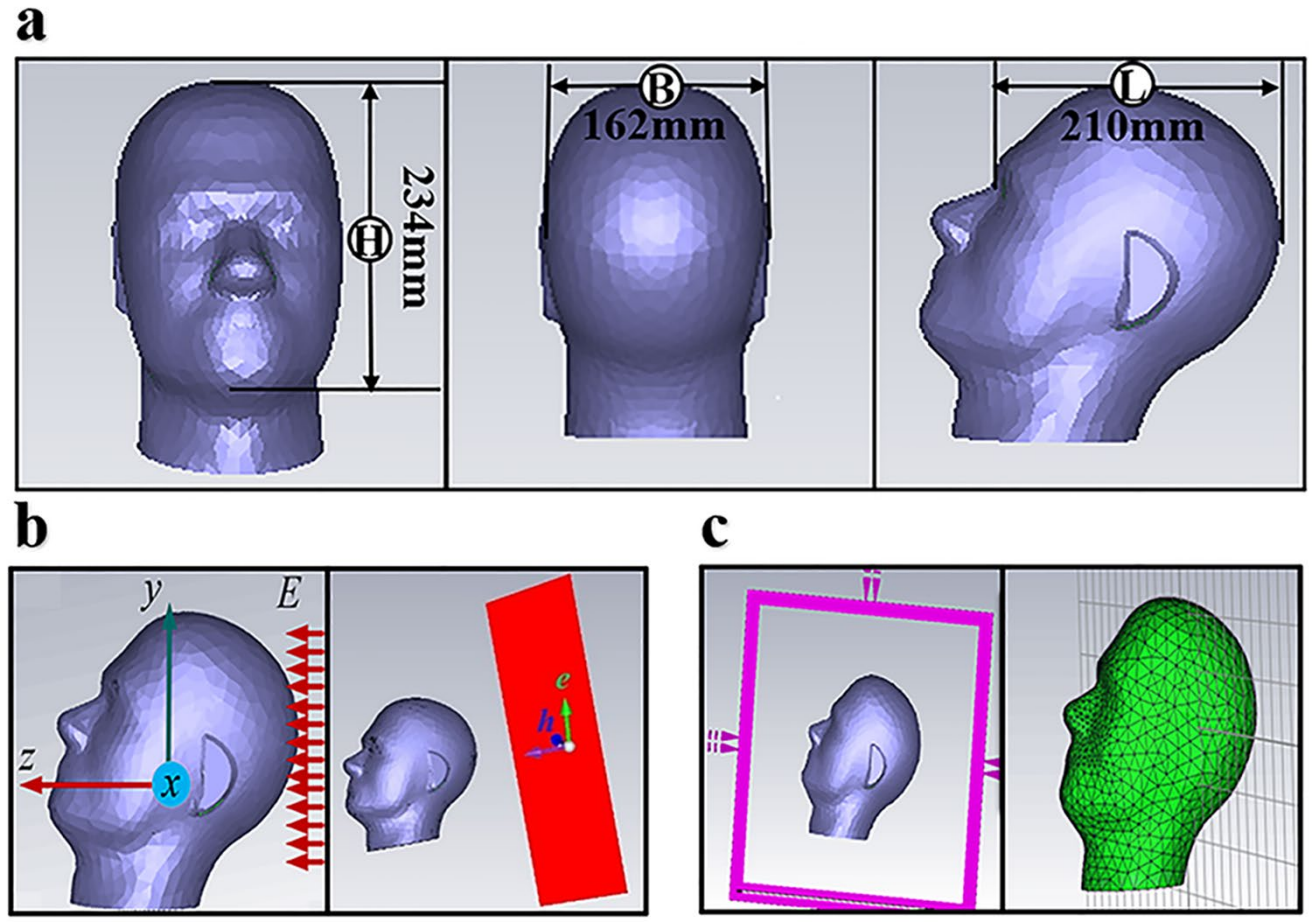
The SAM model and simulation scene. **(a)** Outer shape and size of the SAM model, **(b)** coordinate system definition and the incident wave, **(c)** simulation region and structure partition.

The SAM is a simplified physical model of human head specified by IEEE Standard-1528 and IEC 62209-1, whose shape and selected dimension parameters come from the 90th-percentile anthropometric data corresponding to the adult male head in the United States (US) Army^24^. It consists of two layers: the skull and the brain tissue. The thickness of the skull is 2 mm. The head height, breadth, and length are respectively 234 mm, 162 mm and 210 mm^21^. Figure 1a shows outer shape and size of SAM model. The model skull is constructed from low-permittivity and low-loss dielectric material, while the internal brain tissue has higher dielectric constant and conductivity, and the dielectric constant and the conductivity of the biological tissue change with the frequency. For the sake of simplification, the brain tissue in the head is set to the same electromagnetic parameters, and the dielectric parameters taken here are the values at the frequency of 300 MHz. We set the relative permittivity of the skull $\varepsilon_{o}$ to 5 and the loss tangent $\tan \delta_{o}$ to 0.05, the relative dielectric parameter of the inner brain tissue $\varepsilon_{i}$ to 45, and the conductivity $\sigma_{i}$ to 0.85 S/m.

Generally, the structure of the brain is extremely complex, it can be simplified to a sphere in terms of its geometric shape^25,26^. A model of a spherical brain with different structural parameters has been constructed, which consists of a skull, an interior tissue, and a hole inside as an air cavity. To be as consistent with the SAM as possible, we set the head size to 210 mm, the skull thickness to 2 mm. As far as the dielectric properties of the sphere are concerned, it may be divided into two regions correspondingly. Similarly, considering an EMP of 300 MHz as a reference, the relative dielectric constant and the dielectric loss factor of the sphere skull are respectively $\varepsilon_{o} = 5$, $\tan \delta_{o} = 0.05$, the relative dielectric constant and the conductivity of the brain tissue are respectively $\varepsilon_{i} = 45$, $\sigma_{i} = 0.85{\text{ S/m}}$.

### Simulation scene

The SAM and spherical brain models are used and relevant parameters are set, the process of human brain illuminated by an EMP is numerically simulated, and the field distribution along the Z direction (the posterior to anterior) in the brain is analyzed. The coordinate system definition and the incident wave of SAM simulation are shown in Fig. 1b. The incident field is a Gaussian pulse along the Z axis with a field intensity of 1 V/m, the time-dependent wave equation is given in Eq. (1), among which, $t_{0} = 3.55$, $\tau = 3.42$. The time domain solver is used and the numerical simulation is carried out in the frequency range of 0 to 0.5 GHz. Figure 1c shows the simulation region and model structure partition. The simulation scene of the spherical brain is similar to SAM simulation.

1 $$E_{i} (t) = \exp \left( { - \frac{{4\pi (t - t_{0} )^{2} }}{{\tau^{2} }}} \right) \,$$

## Results

### Dynamic simulation of SAM exposed to EMP of 250 MHz

**Figure 2:**
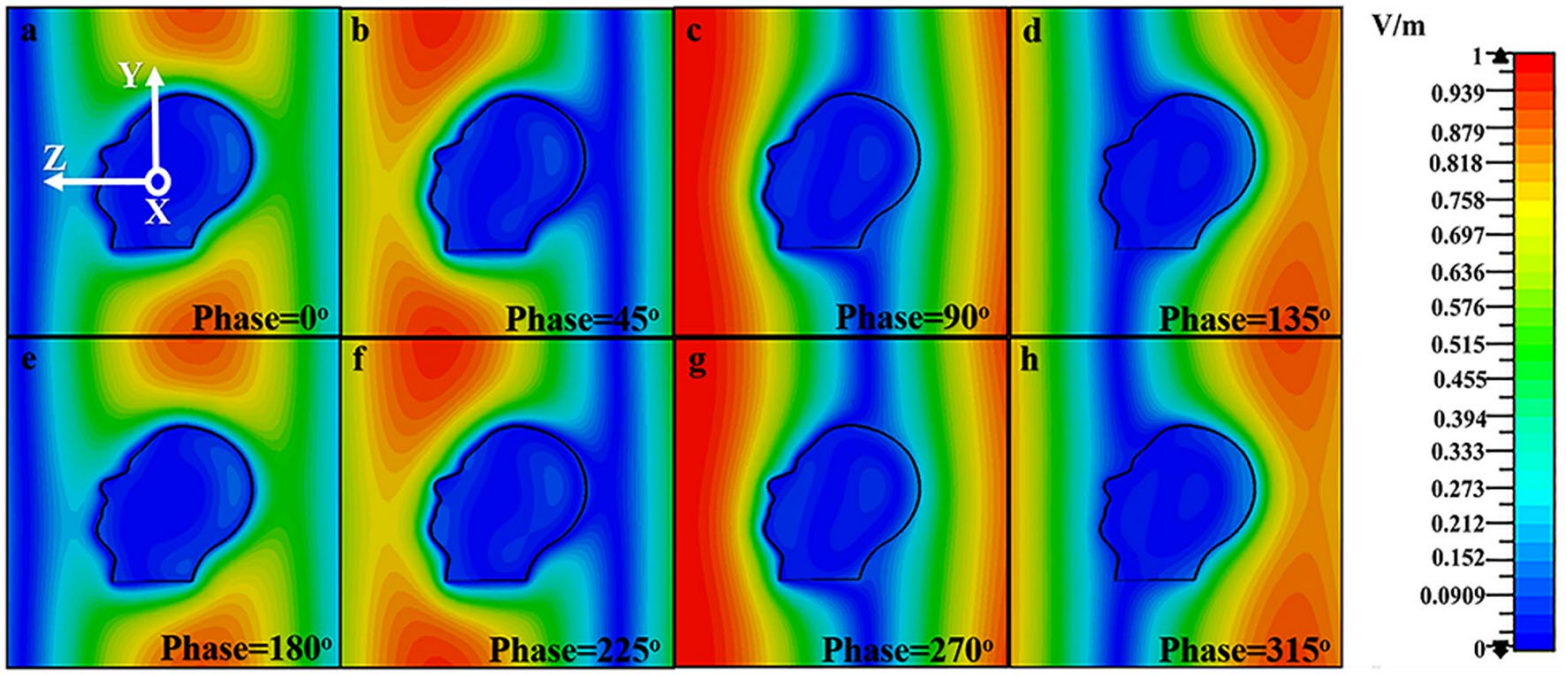
The field distribution under 250 MHz pulse radiation along the Z direction in the simulation region with different phases. **(a)**$Phase = 0^{ \circ }$, **(b)**$Phase = 45^{ \circ }$, **(c)**$Phase = 90^{ \circ }$, **(d)**$Phase = 135^{ \circ }$, **(e)**$Phase = 180^{ \circ }$, **(f)**$Phase = 225^{ \circ }$, **(g)**$Phase = 270^{ \circ }$, **(h)**$Phase = 315^{ \circ }$.

The dynamic simulation of SAM along the Z direction with the center frequency 250 MHz is shown in Fig. 2. The upper right corner is the scaleplate. Figure 2a–h show the field distribution in the simulation region with different phases. It can be seen that after an EMP exposure, there is a great difference in the electric fields of the inner brain and the space surrounding the SAM, and the field distribution in the brain depends heavily on the incident azimuth when exposed to an EMP.

### Influence of individual dielectric parameters difference on electromagnetic distribution

The dielectric parameters of the SAM are adjusted to observe the electromagnetic distribution in the brain along the Z direction. Supplementary Figure S1–S3 show the electromagnetic distribution curves along the Z direction where each figure is a different frequency (0.05 GHz, 0.25 GHz, 0.45 GHz) and each subfigure a, b, c, d respectively indicate each parameter decreased by 20%, 10% and increased by 10%, 20%. As shown in Supplementary Fig. S1a, the horizontal axis of the graph represents the Z direction in SAM simulation, the vertical axis represents the electric field intensity. The center of SAM is at the point ${\rm Z} = 34.853 {\text{mm}}$, the boundary of SAM approximately ranges from −70 mm to 140 mm, and the black curve with square markers ($\varepsilon_{o} = 5$, $\delta_{o} = 2.86$, $\varepsilon_{i} = 45$, $\sigma_{i} = 0.85$) is the control group. The control group represents the SAM simulation with the default dielectric parameters. The following diagrams are similar to those of Supplementary Fig. S1a.

It can be seen from Supplementary Fig. S1–S3 that the field distribution curves almost coincide with those of the control group when $\varepsilon_{o}$ and $\delta_{o}$ are changed, while visibly different from those of the control group when $\varepsilon_{i}$ and $\sigma_{i}$ are changed, especially under the irradiation of high frequency pulse, that is, the field distribution is more sensitive to variations of the brain tissue dielectric parameters than those of the skull dielectric parameters, and the high frequency pulse signal is more sensitive to the variation of dielectric parameters than the low frequency signal.

**Figure 3:**
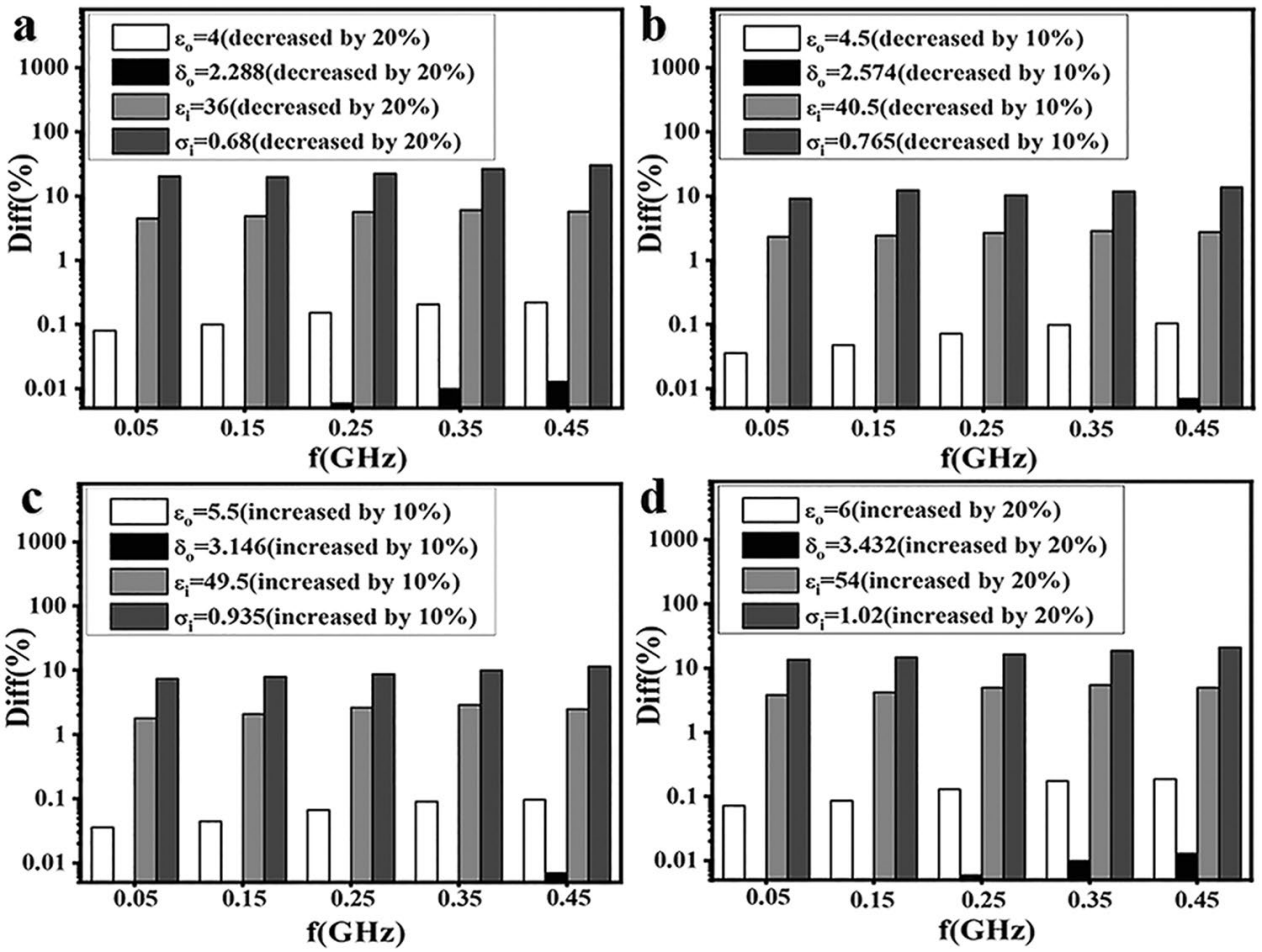
The absolute relative difference between the electric fields in the brain along the Z direction at different frequencies with different adjustment amount of each dielectric parameter. **(a)** Decreased by 20%, **(b)** decreased by 10%, **(c)** increased by 10%, **(d)** increased by 20%.

In addition, in order to gain better understanding of the effect of dielectric parameters variation on the field distribution in the brain, the absolute relative difference between the electric fields in the brain is calculated and analyzed. Figure 3 gives the analysis of the influence of dielectric parameters variation on the average electric field in the brain along the Z direction with different frequencies and a, b, c, d respectively indicate each dielectric parameter is decreased by 20%, 10% and increased by 10%, 20%. As shown in Fig. 3a, the horizontal axis of the graph represents the simulation frequency, the vertical axis represents the absolute relative difference between the electric fields in the brain. Diff (%) indicates the absolute relative difference between the electric fields in the brain of the SAM with a varied dielectric parameter and the control group with respect to the control group. The following diagrams are the same as those of Fig. 3a.

Diff (%) is calculated by the formula $Diff = {{|E - E_{SAM} |} \mathord{\left/ {\vphantom {{|E - E_{SAM} |} {E_{SAM} }}} \right. \kern-\nulldelimiterspace} {E_{SAM} }} \times 100\%$, among which, $E$ is the average electric field in the brain with each frequency and each set of parameters, $E_{SAM}$ is the average electric field in the brain of SAM with a frequency range of 0.05 GHz to 0.45 GHz at intervals of 0.1 GHz. The dielectric parameters of SAM (control group) are $\varepsilon_{o} = 5$, $\delta_{o} = 2.86$, $\varepsilon_{i} = 45$, $\sigma_{i} = 0.85$.

It can be seen from Fig. 3 that the absolute relative differences between the electric fields in the brain are far less than 1% and the maximum values are respectively 0.22% and 0.01% when $\varepsilon_{o}$ and $\delta_{o}$ are changed, while more than 1% and the minimum values are respectively 1.81% and 7.45% when $\varepsilon_{i}$ and $\sigma_{i}$ are changed. Moreover, when dielectric parameters are adjusted, the absolute relative difference between the internal electric fields varies with the frequency. Taking $\sigma_{i}$(decreased by 20%) as an example, the absolute relative difference between the electric fields is under 20% at 0.15 GHz, and over 30% at 0.45 GHz, and it has a substantially difference of over 10%.

The results point out that the $\varepsilon_{o}$ and $\delta_{o}$ have little effect on the field distribution, while the $\varepsilon_{i}$ and $\sigma_{i}$ have a relatively larger influence, that is, the field distribution in the brain exhibits a strong dependence on dielectric parameters of the brain tissue (especially the conductivity of brain tissue), and not the skull. The absolute relative difference between the electric fields in the brain caused by the variation of dielectric parameters will be quite different at different frequencies. It is therefore necessary to consider the frequency when evaluating the variation of field distribution caused by the dielectric parameters of the tissue.

### Influence of individual structure difference on electromagnetic distribution

Considering the influence of individual structure difference, brain models with different head size and skull thickness are simulated. Both the incident EMP and simulation frequency are the same as those of the SAM simulations. Supplementary Figure S4–S6 show the electromagnetic distribution curves along the Z direction where each figure is a different frequency (0.05 GHz, 0.25 GHz, 0.45 GHz) and each subfigure a, b, c, d respectively represent the field distribution with different adjustment amount of head size and skull thickness (decreased by 20%, 10% and increased by 10%, 20%). As shown in Supplementary Fig. S4a, the origin of the coordinate plane is the center of the spherical brain, the horizontal axis of the graph represents the Z direction in spherical brain simulation, and the vertical axis represents the electric field intensity. The black curve with square markers ($R_{o} = 105{\text{ mm}}$,${\rm T} = 2 {\text{mm}}$) indicates the control group. The control group represents the spherical brain simulation with the default dielectric parameters. The following diagrams are similar to those of Supplementary Fig. S4a.

It can be seen from Supplementary Fig. S4–S6 that the field distribution curves almost coincide with those of the control group with variation of ${\rm T}$, while visibly different from those of the control group with variation of $R_{o}$, especially under the irradiation of high frequency pulse, that is, the field distribution is more sensitive to variations of the head size than those of the skull thickness, and the high frequency signal is more sensitive to the variation of structural parameters than the low frequency signal.

**Figure 4:**
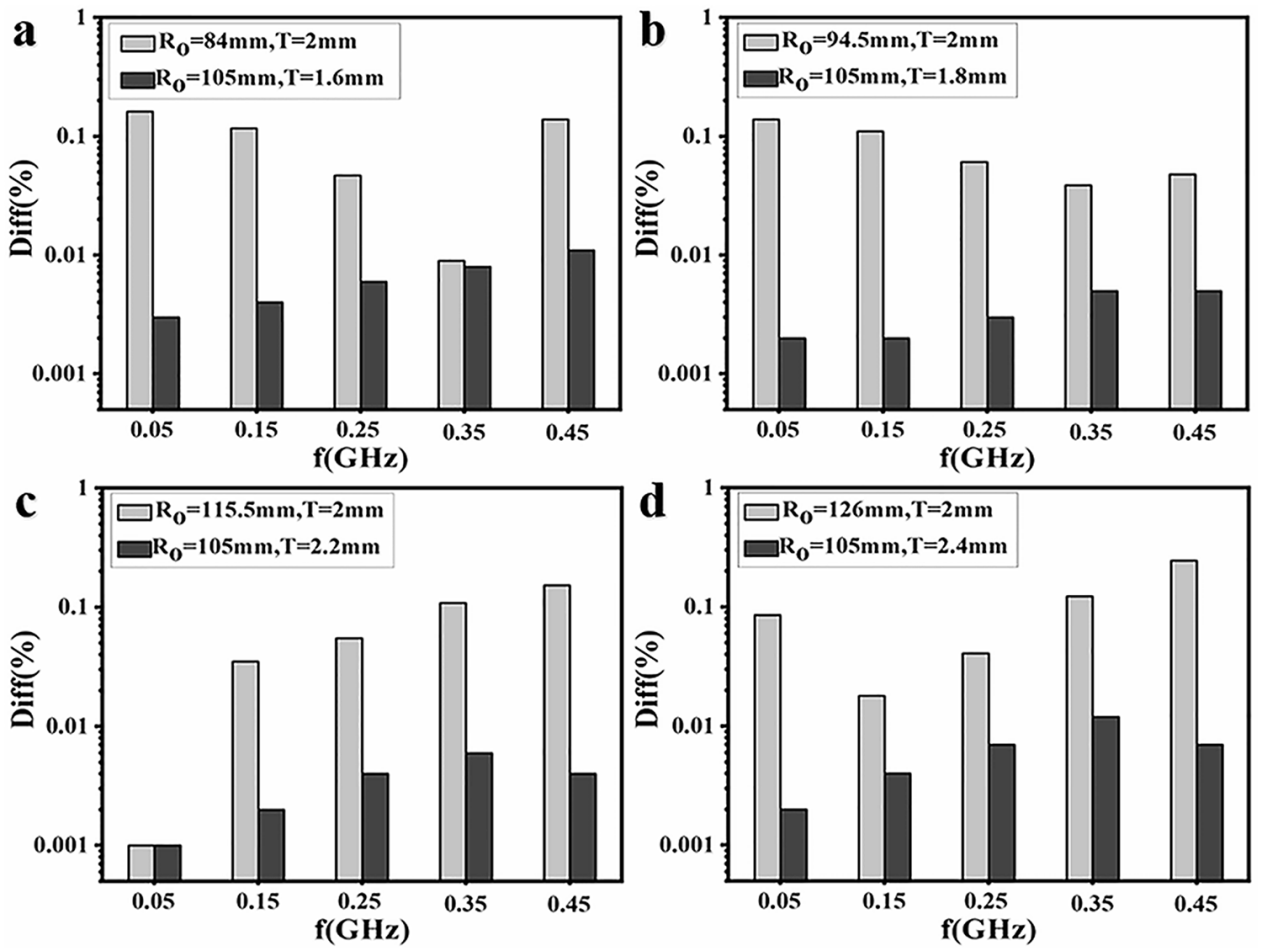
The absolute relative difference between the electric fields in the brain along the Z direction at different frequencies with different adjustment amount of each structural parameter. **(a)** Decreased by 20%, **(b)** decreased by 10%, **(c)** increased by 10%, **(d)** increased by 20%.

Also, in order to show the influence of structure difference on the field distribution in the brain more intuitively, the absolute relative difference between the electric fields in the brain is calculated and analyzed with different frequencies and different structural parameters. Figure 4 shows the analysis of the influence of varying structural parameters on the average electric field in the brain along the Z direction with different frequencies and a, b, c, d respectively indicate each structural parameter is decreased by 20%, 10% and increased by 10%, 20%. As shown in Fig. 4a, the horizontal axis of the graph represents the simulation frequency, the vertical axis represents the absolute relative difference between the electric fields in the brain. Diff (%) indicates the absolute relative difference between the internal electric fields of standard spherical brain (SSB) with a varied structural parameter and the control group with respect to the control group. The following diagrams are the same as those of Fig. 4a.

Diff (%) is calculated by the formula $Diff = {{|E^{\prime} - E_{SSB} |} \mathord{\left/ {\vphantom {{|E^{\prime} - E_{SSB} |} {E_{SSB} }}} \right. \kern-\nulldelimiterspace} {E_{SSB} }} \times 100\%$, among which, $E^{\prime}$ is the average electric field in the brain with each frequency and each set of parameters, $E_{SSB}$ is the average electric field in the brain of SSB with a frequency range of 0.05 GHz to 0.45 GHz at intervals of 0.1 GHz. The structural parameters of SSB (control group) are $R_{o} = 105{\text{ mm}}$,${\rm T} = 2 {\text{mm}}$*.*

It can be seen from Fig. 4 that the absolute relative differences between the electric fields are far less than 1% when the size of the head is changed and broadly under 0.01% when the thickness of the skull is changed. Furthermore, when the structural parameters are adjusted, the differences of the absolute relative difference between the electric fields in the brain at different frequencies were observed. Taking $R_{o}$(increased by 20%) as an example, the absolute relative difference is under 0.02% at 0.15 GHz, while over 0.2% at 0.45 GHz, and it has a relatively considerable difference of over 1%.

The results point out that no apparent relation between the skull thickness and the field distribution, and the electromagnetic field distribution in the brain is mainly related to the size of the head. The absolute relative difference between the electric fields in the brain caused by the variation of head size will be a little bit different at different frequencies and it should not be neglected when assessing the variation of field distribution caused by the structural parameters of the model.

### The multi-section electric field in the brain exposed to an EMP

**Figure 5:**
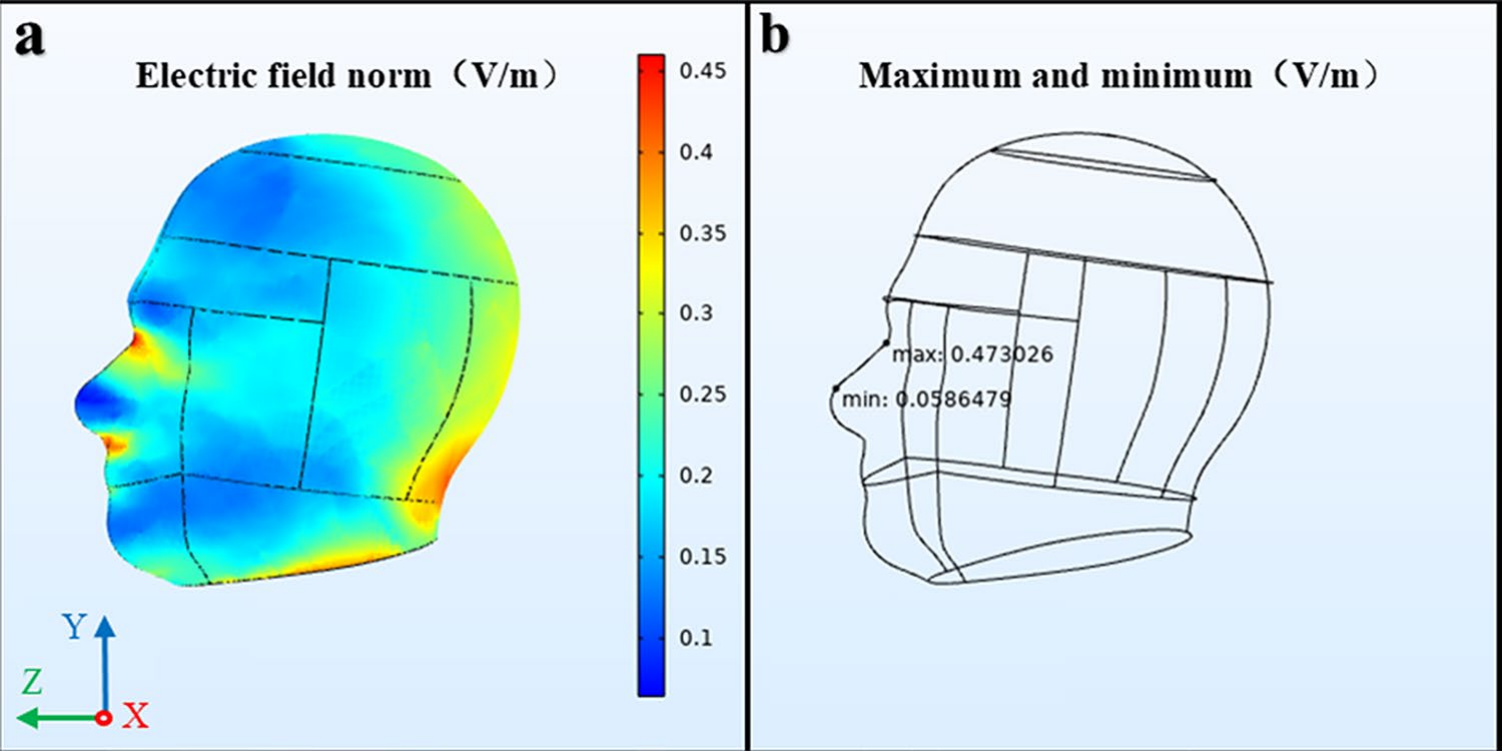
The multi-section electric field in the brain exposed to an EMP at 300 MHz. **(a)** The electric field norm, **(b)** the maximum and minimum electric field.

To appreciate the magnitude of the transmitted EMP in the SAM, we consider a typical EMP with peak electric field strength of 1 V/m, and the propagation direction along the Z axis (mentioned in the simulation scene). The three-dimension multi-section electric field in the human brain after an EMP exposure with the frequency 300 MHz is shown in Fig. 5. Figure 5a shows the multi-section electric field norm in the brain and Fig. 5b displays the positions corresponding to the maximum and minimum electric field.

Table 1 provides the maximum, minimum, and average electric fields in the brain exposed to EMPs in the frequency range of 10–500 MHz, which denoted by E_min_, E_max_, and E_a_ respectively.

**Table 1:** The maximum, minimum, and average electric fields.

Frequency(MHz)	E_min_ (V/m)	E_max_ (V/m)	E_a_ (V/m)
10	3.46E−5	2.34E−2	8.03E−3
50	3.78E−4	1.16E−1	4.06E−2
100	1.46E−3	2.38E−1	8.52E−2
200	4.29E−2	4.95E−1	1.59E−1
250	5.27E−2	5.31E−1	1.84E−1
300	5.86E−2	4.73E−1	2.06E−1
400	3.14E−2	4.80E−1	2.14E−1
500	1.69E−2	4.81E−1	2.01E−1

As obtained from Fig. 5, the transmitted field is the highest at the surface of the SAM head and it decreases promptly with increasing distance into the model when exposed to an EMP of 300 MHz, and the peak transmitted pulse amplitude in the SAM model is about 0.47 V/m. It can be seen from Table 1 that when exposed to an EMP, the magnitude of the average electric field in the human brain is about 1/100–1/5 of the incident field within the studied frequency range.

## Discussion

In this paper, the electromagnetic distribution in the human brain is obtained by CST simulation. The SAM simulation results show that the average electric field in the brain ranges from 1/100 to 1/5 of the incident field after an EMP exposure. And both simulation results of the SAM and spherical brain models show that the distribution of the electric field in the human brain mainly depends on the size of the head and dielectric parameters of the brain tissue. The degree of variation in the brain field distribution caused by variations of individual tissue dielectric parameters and head size will be different at different frequencies, the degree of variation increases with frequency; that is, high-frequency signals within the studied frequency range are more sensitive to the individual differences. On the part of individual dielectric parameters, the electromagnetic distribution in the human brain mainly depends on the dielectric parameters of the brain tissue (especially the conductivity of brain tissue), the skull dielectric parameters have little effect on the internal electromagnetic distribution. In terms of the individual structure, the field intensity in the brain is greatly affected by the size of the head, while the thickness of the skull exerts little influence. These results indicate that the intracerebral field distribution is more sensitive to variations of the brain tissue dielectric parameters and head size than those of the skull dielectric parameters and skull thickness.

As a whole, the average electric filed in the human head is about 1/100 to 1/5 of the incident field intensity within the studied frequency range (10–500 MHz), which will provide theoretical basis for the mechanism study of EMP-induced BBB opening in the future research. In addition, compared with adjustments of input parameters themselves, individual differences have little effects on the field distribution in the human brain; and thus, it is reliable to establish the connection between the internal electric field and the effect in the studied frequency range, which will provide some guiding significance for the construction of brain models and the setting of electromagnetic parameters in the future when performing similar research. Furthermore, it will offer a dataset that is useful for UQ and the UQ data from these simulations can inform further studies that utilize high-fidelity models. A follow-on study might utilize the high-fidelity phantoms to add to these uncertainty estimates.

## Supplementary Information

Supplementary Figures.

## Data Availability

The datasets generated or analysed during the current study are available from the corresponding author on reasonable request.
