# Supplementary material for: Numerical simulation and analysis of effects of individual differences on the field distribution in the human brain with electromagnetic pulses
Source: Sci Rep. 2021 Aug 13;11:16504. doi: 10.1038/s41598-021-96059-3 (PMC8363612; doi:10.1038/s41598-021-96059-3)
Supplement: Supplementary file 1 — Supplementary Figures. [file 41598_2021_96059_MOESM1_ESM.pdf]

Supplementary Information

Numerical simulation and analysis of effects of individual differences on the field distribution in the human brain with electromagnetic pulses

Shan Wang[1], Zhongguo Song[1], Huiping Li[1], Guozhen Guo[2], Xiaoli Xi[1,]*

[1] Faculty of Automation and Information Engineering, Xi'an University of Technology, Xi'an, 710048, China

[2] Department of Radiation Biology, Air Force Medical University, Xi'an, 710032, China

** xixiaoli@xaut.edu.cn*

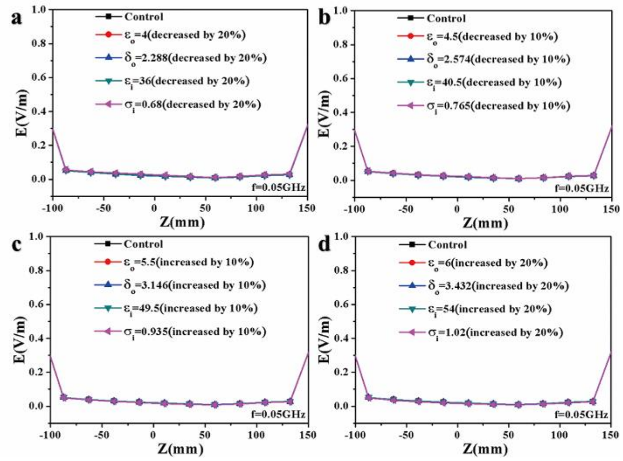

Figure S1. The electric field distribution curves along the Z direction at 0.05 GHz with different adjustment amount of each dielectric parameter. (a) Decreased by 20%, (b) Decreased by 10%, (c) Increased by 10%, (d) Increased by 20%.

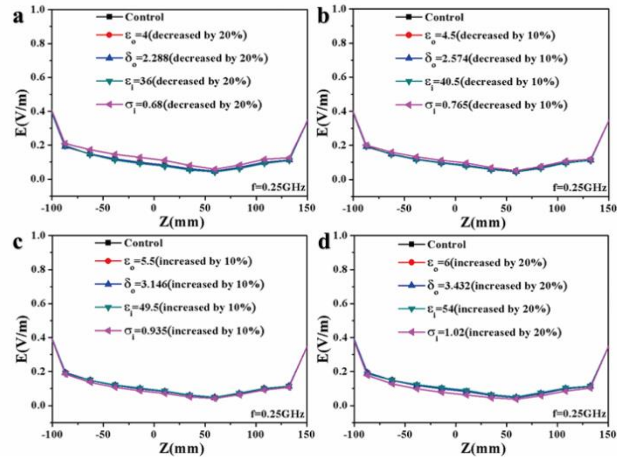

Figure S2. The electric field distribution curves along the Z direction at 0.25 GHz with different adjustment amount of each dielectric parameter. (a) Decreased by 20%, (b) Decreased by 10%, (c) Increased by 10%, (d) Increased by 20%.

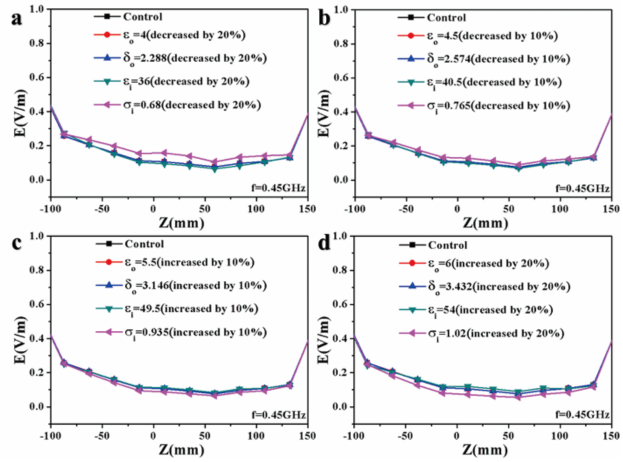

Figure S3. The electric field distribution curves along the Z direction at 0.45 GHz with different adjustment amount of each dielectric parameter. (a) Decreased by 20%, (b) Decreased by 10%, (c) Increased by 10%, (d) Increased by 20%.

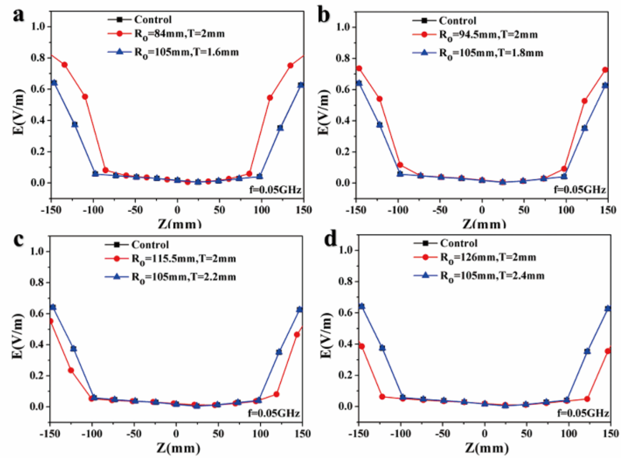

Figure S4. Distribution curves of the electric field along the Z direction at 0.05 GHz with different adjustment amount of R_o *and* T *. (a) Decreased by 20%, (b) Decreased by 10%, (c) Increased by 10%, (d) Increased by 20%.*

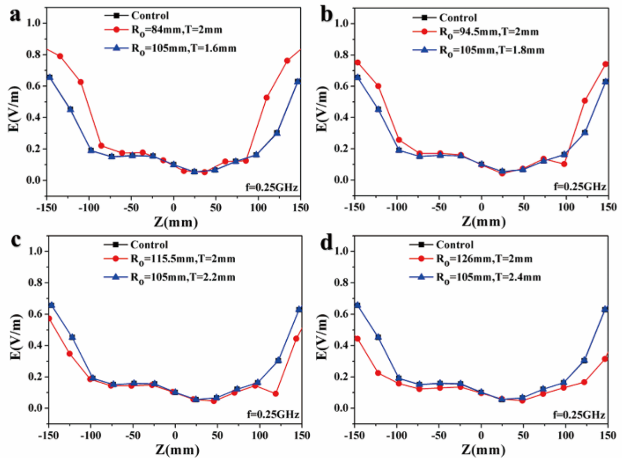

Figure S5. Distribution curves of the electric field along the Z direction at 0.25 GHz with different adjustment amount of R_o *and* T *. (a) Decreased by 20%, (b) Decreased by 10%, (c) Increased by 10%, (d) Increased by 20%.*

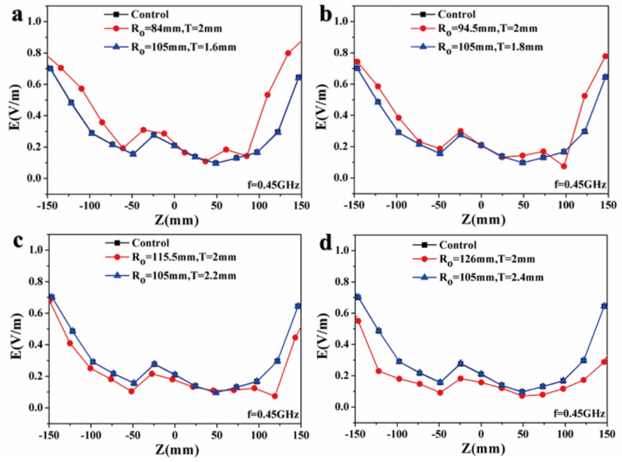

Figure S6. Distribution curves of the electric field along the Z direction at 0.45 GHz with different adjustment amount of R_o *and* T *. (a) Decreased by 20%, (b) Decreased by 10%, (c) Increased by 10%, (d) Increased by 20%.*
